# Telemedicine as an unexpected catalyst during and beyond the COVID-19 Pandemic

**DOI:** 10.3126/nje.v12i1.42459

**Published:** 2022-03-31

**Authors:** Mahendra Kumar, Pushpa Rani, Binal Joshi, Roop Kishor Soni, Anita Kumari, Kusum K Rohilla

**Affiliations:** 1 Stroke Team Coordinator, Department of Neurology, PGIMER, Chandigarh, India; 2 All India Institute of Medical Sciences, Rishikesh, India; 3 Manikaka Topawala Institute of Nursing Charotar University of Science and Technology, India; 4 PGIMER, Chandigarh, India; 5 Captain at Military Nursing Core, Indian Army Chandigarh, India; 6 Ph.D. Scholar (Palliative Care), All India Institute of Medical Sciences, Rishikesh, India

**Keywords:** Coronavirus Infections, Disease Outbreak, Pandemics, Patient Care Management, Telemedicine

## Abstract

Telemedicine that also known as the practice of medicine at a distance whereby information technology is used to ensure the delivery of medical care services. Telemedicine is not a new concept in the world and India.Indian Space Research Organization (ISRO) started telemedicine in India during year 2001 as a pilot project and in year 2005 Ministry of Health and family welfare started full time program of telemedicine by connecting all major health institutions. Telemedicine is connecting people across border and culture. The need-based changes are coming in telemedicine sectors such as smart apps, involvement of private sector players and high intensity internet connections reaching to rural areas and difficult demographic locations. During Covid-19 pandemic telemedicine benefited people by supplying health information and consultation without breaching them without breeching physical contact restrictions. The ease of access to telemedicine applications, its low cost, and the lack of infrastructure requirements propelled to become the top choice in these dayswhere physical distancingconsidered the aforementioned, thus we can conclude that telemedicine is promising tool.

## Background

Health for all India’s mission is experiencing a major roadblock due to the lack of availability of an adequate number of doctors and nurses, especially in the underprivileged areas of the country due to the misdistribution of resources [1]. In India, the most recent data shows a doctor-to-patient ratio of about 0.62:1000 people, which is far much lower than the recommended 1:1,000 as per World Health Organization (WHO) [2, 3]. COVID-19 persistent spread worsens the situation further by creating an acute shortage of health care professionals, especially qualified doctors [4].

Pandemics pose challenges to any health care systems and restrict face-to-face physician-patient communication. The emergence of the coronavirus disease in laterpart of 2019 has changed our lives drastically. To flatten the curve of COVID-19, social distancing restrictions and lockdowns have been announced by civic agencies globally. The routine outpatient department (OPD) has not been functioning to full capacity in physical mode at most hospitals and has led to an increasing dependence on virtual medical visits to their patients [5]. World Health Organization and the Centers for Disease Control also suggest encouraging telemedicine to provide a safe and effective alternative to physical visits.

During pandemic, health systems are under pressure to weigh aforementioned limitations to meet increasing demands. Social distancing restrictions in COVID pandemic have provided a unique opportunity for the widespread use of telemedicine. There has been an exponential increase in usage of telemedicine in COVID-19 pandemic. Therefore to provide uninterrupted health coverage to every corner of society, telemedicine is the answer to bypass and break the COVID-19 infection chain [6].

### What is Telemedicine?

Telemedicine is part of telehealth. The word “Tele” meaning “distance” and “Medicine” meaning “to heal”. Another synonymous of telemedicine by Time Magazine as “healing by wire” [7]. Telemedicine also refers to the practice of medicine at a distance whereby information technology is used to ensure the delivery of medical care services. By using mobile phones, laptops and computers, healthcare providers and doctors can communicate with their patients virtually and write prescriptions or follow-ups [8,9].

### Advantages of Tele medicine

As a combination of both technologies and devices telemedicine supports health care centers to assess the health status of people sitting from a far overcoming geographical barriers and connecting users remotely. Telemedicine also reduced cost and effort as well as time, as patients does not need to travel long distances to get consultation as well as treatment.Thus, family and caregivers’inconvenience are also reduced significantly [10].

Newer technology has enabled the digitization of records, to provide better coverage to privacy. Telemedicine can help in the decongestion of hospitals for routine visits of the patient such as regular or routine check-ups or continuous monitoring,and nonsurgical treatment. Thus, this can reduce the burden on health centers already crumbling due to the pressure of the current pandemic. Before COVID-19 pandemic, usage of telemedicine in USA was about 8% only, since announcement of the COVID-19 pandemic a stark increase of 683% usage of telemedicine [7].

### Telemedicine services in India

Telemedicine is not novice concept in the world along with India. WHO defined telemedicine as “healthcare services delivery to a distance” [11]. Continuity in follow-up through telemedicine can help patients to better manage their disease’s condition and adhere to their medication regimens? Reference Telemedicine works are either real-time or store-and-transfer models. This depends on data relay, availability of network, and infrastructure facilities. In India, most common model used for telemedicine networks is hub-and-spoke model, where the hub is typically a tertiary level healthcare center like medical college hospitals and spokes are the peripheral health facilities such as sub-centers, community health centers, primary health care centers, and district hospitals [12]. India has one of the cheapest costs of data available in mobile networks. Various type of free software currently available, such as Google meet, Skype, Zoom, WhatsApp and Webex. An availability of good camera quality mobile phones allowsproper teleconsultation evaluation of any patient.

### Experts’ and users opinions about Telemedicine

The recent updates in telemedicine practice guidelines, massive advancement of internet infrastructure and internet speed, improved information storage databases, made telemedicine stress-free and user-friendly. Importantly many studies reported that patients, doctors, and clinical experts are in favor of telemedicine due to a sense of comfort at being home, and interaction with physician increasing acceptance amongst patients [13]. Another large benefit of using telemedicine is short triage, which can be done even before the arrival of the patient at the health care center. In lower-income countries like India, telemedicine effectively reduces transport costs and health consultation expenses.

Additional benefits of telemedicine are that they do not requirement of multi-infrastructure set-up, low-cost operations, all specialist availability on a single platform and it save time and efforts. Furthermore, in remote areas, telemedicine is a gift and the distance factor is completely wiped out so that patients from anywhere can receive specialist consultations irrespective of the distance.

Another study form Poland reported that the participants acknowledge the positive impact of telemedicine. They found it more comfortable to share their problems with their doctors [14]. Paucity of researches examined effectiveness of telemedicine and their finding suggested that it reduced both hospital admissions and cost of treating any patients [15]. Another study from India by Dash S, et al also highlighted positive role of telemedicine during COVID-19 pandemic [16].

### Constraints to Telemedicine

Apart from the many benefits and advantages of telemedicine, there are some limitations too. Many doctors believe that technical issues are the main barrier encounteredin telemedicine. In the absence of a physical examination, doctors are not 100% sure about the initial diagnosis because any important sign or symptom may be missed.

Another common concern with telemedicine is the lack of effective interaction between doctor and patient and missing the “human touch” [7]. Furthermore there is an urgent need to improve awareness about telemedicine and strong technical support to providers and end-users for a sound experience of consultation [17].

A study sharing experience of telemedicine during pandemic from Madhya Pradesh conducted by Saxena S, et al. India also reveals that expansion of telemedicine is still a concern and people prefer physical outpatient department (OPD) than tele mode [18].

The risk of a communication gap or language can be a barrier. People do not want to pay high consultation fees for online consultations, which is an odd but true fact. Many people found this expensive as, without any physical visit, there is no physical examination that is as accurate as that of the physical model.

### For support and cons of Telemedicine

- **For Support of telemedicine**
  - It promote social distancing and reduce chance of hospital burn infection
  - Promote health care delivery in remote and far ahead areas
  - Open new door for opportunities in health care
  - Provide better privacy and solve mobility concerns of patients who found it difficult to reach health centers
  - Reduce cost and investment
- **Cons of Telemedicine**
  - Required high end internet connectivity
  - Consenting and ethical issue in some cases
  - Need to improve tele infrastructure
  - Patient satisfaction is still a concern.

The world has not yet got rid of the pandemic and many phases are to come. Today telemedicine is becoming popular at a rapid pace. Telemedicine has been found to be the mainstay of patient care during the current pandemic. Telemedicine helped to provide critical patient follow-ups continuity and avoid exposure to health systems and healthcare workers.
